# Diva Reduces Cell Death in Response to Oxidative Stress and Cytotoxicity

**DOI:** 10.1371/journal.pone.0043180

**Published:** 2012-08-15

**Authors:** Nicole Suyun Liu, Xiaoli Du, Jia Lu, Bei Ping He

**Affiliations:** 1 Department of Anatomy, Yong Loo Lin School of Medicine, National University of Singapore, Singapore, Singapore; 2 Duke-NUS Graduate Medical School Singapore, Singapore, Singapore; 3 Defence Medical and Environmental Research Institute, DSO National Laboratories, Singapore, Singapore; University of Texas Health Science Center at San Antonio, United States of America

## Abstract

Diva is a member of the Bcl2 family but its function in apoptosis remains largely unclear because of its specific expression found within limited adult tissues. Previous overexpression studies done on various cell lines yielded conflicting conclusions pertaining to its apoptotic function. Here, we discovered the expression of endogenous Diva in PC12 neuronal-like cell line and rat bone marrow mesenchymal stem cells (BMSCs), leading to their utilisation for the functional study of Diva. Through usage of recombinant Fas ligand, hydrogen peroxide, overexpression and knock down experiments, we discovered that Diva plays a crucial pro-survival role via the mitochondrial death pathway. In addition, immunoprecipitation studies also noted a decrease in Diva’s interaction with Bcl2 and Bax following apoptosis induced by oxidative stress. By overexpressing Diva in BMSCs, we had observed an increase in the cells’ capacity to survive under oxidative stress and microglial toxicity. The result obtained from our study gives us reason to believe that Diva plays an important role in controlling the survival of BMSCs. Through overexpression of Diva, the viability of these BMSCs may be boosted under adverse conditions.

## Introduction

The B-cell lymphoma-2 (Bcl2) family of proteins play key roles in initiating the mitochondrial death pathway. Through complex interaction between these members, the mitochondrial outer membrane permeability (MOMP) can be altered [1]. Members of this family can be broadly classified under anti-apoptotic proteins, effector pro-apoptotic proteins, or BH3 only pro-apoptotic proteins. With intricate interactions occurring between these 3 classes of proteins, the survival status of a cell is ultimately dictated. Anti-apoptotic family members include Bcl2, Bcl-xL, Bcl-w and Mcl-1 which serve to protect cells against death [2]. On the other hand, effector pro-apoptotic members such as Bax and Bak directly engage the mitochondrial membrane to induce apoptosis [3], [4]. The last group of BH3 only proteins can be sub-divided into sensitizers, derepressors and direct activators [5]. Sensitizers are not capable of inducing apoptosis but they work to lower the threshold for Bax and Bak activation [6]. Derepressors on the other hand can release a direct activator from anti-apoptotic protein sequestration, thereby allowing the direct activator to promote MOMP [5], [6]. Direct activators like Bid and Bim can bind to both anti-apoptotic and effector pro-apoptotic proteins, causing Bak/Bax oligomerization and MOMP to occur [3], [7], [8].

Diva is a relatively understudied Bcl2 family member whose function in apoptosis remains unclear. Through earlier studies, it was found that the expression of Diva initially exists ubiquitously in murine embryonic tissues. However as mice develop, the distribution of Diva becomes limited to the adult ovary and testis [9]. In humans, the homologue of Diva is termed as Nrh and this protein is expressed in the liver, kidney and ovaries [10]. In an attempt to perform a sequence alignment of Diva against other Bcl2 members, the existence of conserved BH1, BH2, BH4 and hydrophobic C-terminal domains had been identified [9], [11]. However, results also found that the BH3 region of Diva lacks critical residues that are typical of other Bcl2 family members [9]. This discrepancy contributed to the confusion regarding the true apoptotic nature of Diva. Several groups had attempted to overexpress this protein in Hela cells, 293 cells, Cos-7 cells and cancer cells that do not contain endogenous Diva, only to show conflicting results pertaining to its role in apoptosis. Despite overexpressing Diva within the same cell lines, some groups had concluded it as a pro-apoptotic protein while others found it to be anti-apoptotic [9], [11], [12], [13]. To determine the identity of Diva’s interacting partners, immunoprecipitation studies were also carried out extensively and results picked out a number of associating partners including Bcl2, Bax, Bak and Apaf-1 [9], [11], [14], [15]. Yet even these data were baffling as different groups yielded contrasting results.

Given the large repertoire of Bcl2 proteins, functional redundancy among members exists [16]. An example can be seen in viable Bcl2 null mice which still completed embryonic development despite displaying abnormalities [17]. Bim deletion on the other hand could only transiently protect cells from neuronal apoptosis, as its pro-apoptotic role was partially replaced by other BH-3 only members that functioned upstream of the Bax-mediated cytochrome C release [18]. With Diva being overexpressed to varying extents within similar cell lines, it is possible that the differing compensational effect by other Bcl2 members could have resulted in the difference in apoptotic outcomes [10], [11]. To top that up, the lack of Diva in these cell lines used could have also contributed to the discrepancy in associating partners being identified. Tissue specific expression of Bcl2 members is common and an example can be seen in anti-apoptotic A1 protein which displays restricted localisation within the embryonic liver, heart, nervous system, and cartilaginous primordial [19]. In addition, restricted expression of Bok within rat ovary, testis and uterus had also been reported [20]. Different proteins in specific sites would require interaction with selected proteins within the location. As such, the use of cell lines which do not harbour endogenous Diva might not enable accurate study of Diva’s native partners. Under such circumstances, our team had decided to utilise cells which endogenously express Diva. In this way, we not only are able to observe the reciprocal effects of Diva manipulation, but we can also study the molecular pathway of Diva and its association with native proteins.

In earlier studies conducted, we had identified the presence of Diva mRNA and protein in bone marrow derived mesenchymal stem cells that were isolated from one-month-old male *Sprague–Dawley* rats. This led to the belief that Diva might actually play a role in controlling the survival of these stem cells. It had been previously shown that mesenchymal stem cells (MSCs) possess functional apoptotic pathways that alter according to the stage of MSC differentiation [8]. While undifferentiated MSCs remain more resistant to apoptosis, the differentiated cells become highly susceptible to cell death. Furthermore, cultured MSCs exhibit strong survival capacities in culture but as soon as they get transplanted, they die upon exposure to pro-inflammatory conditions, hypoxia or anoikis [9], [10], [11], [12]. Here, we utilised recombinant Fas ligand (FasL), hydrogen peroxide (H_2_O_2_), Diva overexpression, and Diva silencing to study the apoptotic pathway of Diva. Our results confirmed the anti-apoptotic function of Diva as its overexpression strongly resisted cell death, and its loss led to large scale apoptosis via the mitochondrial pathway. Through interaction studies, we had also identified Bcl2 and Bax as Diva’s native interacting partners that might work together to balance apoptosis. By overexpressing Diva in BMSCs, we were able to enhance the survival of the stem cells in response to oxidative stress and microglial cytotoxicity. With this, we not only hope to shed some light on the function and molecular pathway of Diva, but also to present an alternative way in which the viability of BMSCs could be increased for transplantation in future.

## Results

### Diva Acts through the Intrinsic Mitochondrial Death Pathway

**Figure 1:**
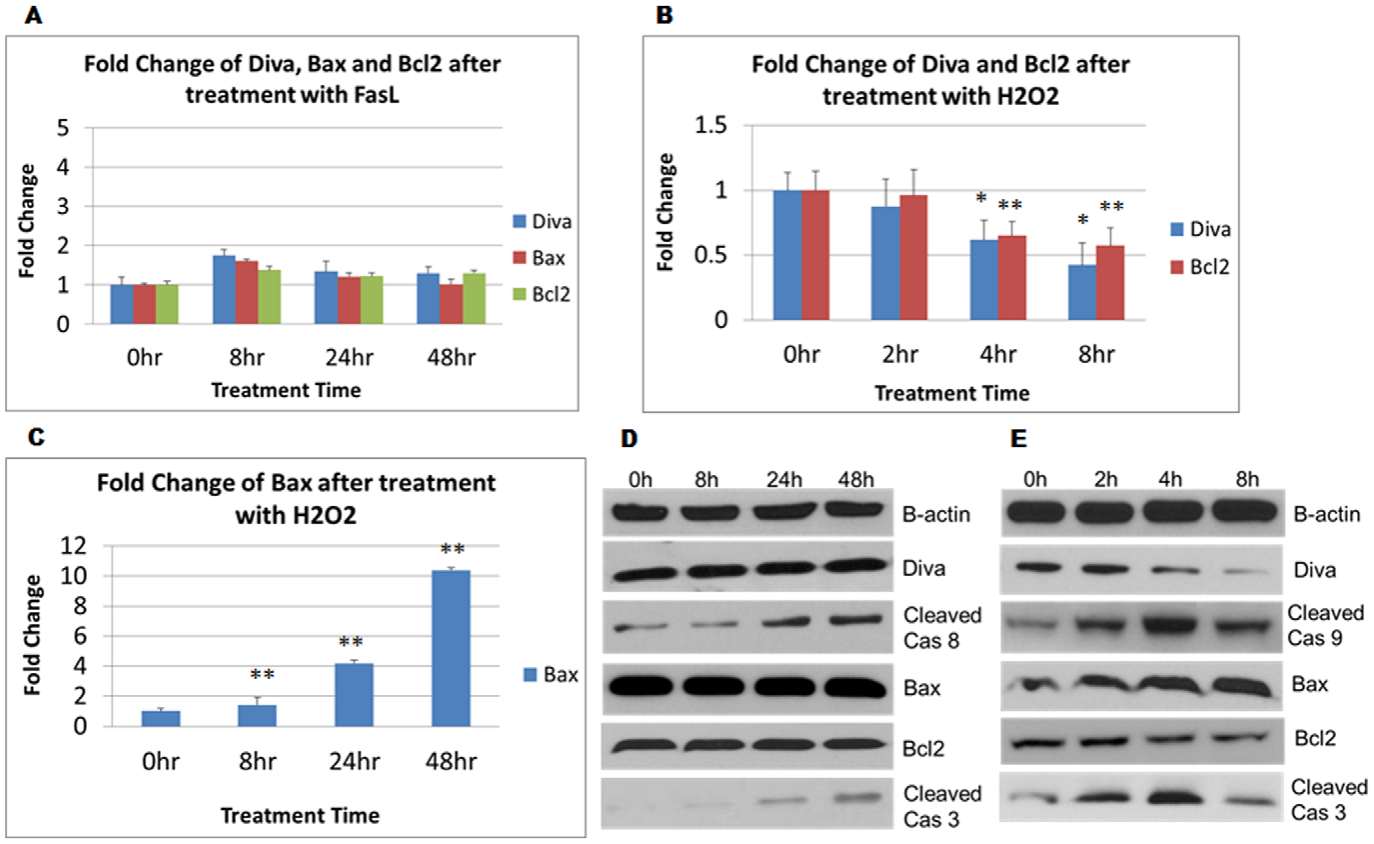
Diva acts through the intrinsic mitochondrial death pathway. a PC12 cells were harvested for total RNA isolation and quantitative real-time PCR to determine the changes in mRNA expression levels of Diva, Bcl2 and Bax following 100 ng/ml of FasL treatment. RNA was extracted 0 hr, 8 hrs, 24 hrs and 48 hrs after treatment and the fold changes were calculated with GAPDH as the internal control. **b** Fold changes of Diva and Bcl2 after addition of 30 nM of H_2_O_2_ for 0 hrs, 2 hrs, 4 hrs and 8 hrs. **c** Fold change of Bax after 30 nM of H_2_O_2_ treatment across 8 hrs. Bar graphs represent mean ± SD from three independent experiments. *p<0.05, **p<0.01. **d** PC12 cells were harvested to determine the protein expression levels of Diva, Cleaved Caspase 8, Bax, Bcl2 and Cleaved Caspase 3. Western blot was conducted on the total protein extracted following 100 ng/ml of FasL treatment for 0 hrs, 8 hrs, 24 hrs and 48 hrs. **e** PC12 cells were harvested to determine the protein expression levels of Diva, Cleaved Caspase 9, Bax, Bcl2 and Cleaved Caspase 3. Western blot was conducted on the total protein extracted following 30 nM of H_2_O_2_ treatment for 0 hrs, 2 hrs, 4 hrs and 8 hrs. B-actin was used as the loading control.

In order to investigate the pathway in which Diva acts through, we utilised 100 ng/ml of recombinant FasL and 30 nM of H_2_O_2_ to induce cell death in PC12 via the receptor and mitochondrial death pathways respectively. The death receptor pathway involves the binding of FasL to the corresponding Fas receptor expressed on the cell surface. Subsequent activation of initiator caspase 8 aids in amplification of the cascade [21]. On the other hand, the mitochondrial pathway involves the interplay between anti and pro-apoptotic Bcl2 members that govern the formation of the apoptosome. Once formed, the apoptosome enables the autoprocessing of capase 9 that will in turn cleave effector caspase 3 [22]. As indicated in figure 1a, no evident fold change was observed in Diva, Bcl2 or Bax level despite exposure of PC12 to FasL for up to 48 hrs. Translationally, the western blot also revealed no change in Diva protein expression following FasL induced apoptosis. The fact that caspase 8 and 3 were activated in the process confirmed that the death receptor pathway was activated (Fig. 1d). In contrast to FasL, figure 1b showed a significant 60% and 50% decline in Diva and Bcl2 mRNA expressions respectively by the end of an 8 hr exposure to H_2_O_2_. Bax on the other hand had risen 10 folds after 8 hrs (Fig. 1c). Similarly, protein levels of Diva and Bcl2 were shown to decrease gradually under prolonged oxidative stress. Bax, cleaved caspase 9 and cleaved caspase 3 levels on the other hand had increased following H_2_O_2_ induced mitochondrial cell death (Fig. 1e). Clearly, Diva’s role was more prominent in mitochondrial oxidative stress as compared to receptor induced death.

### Bax and Bcl2 are Associating Partners of Diva

**Figure 2:**
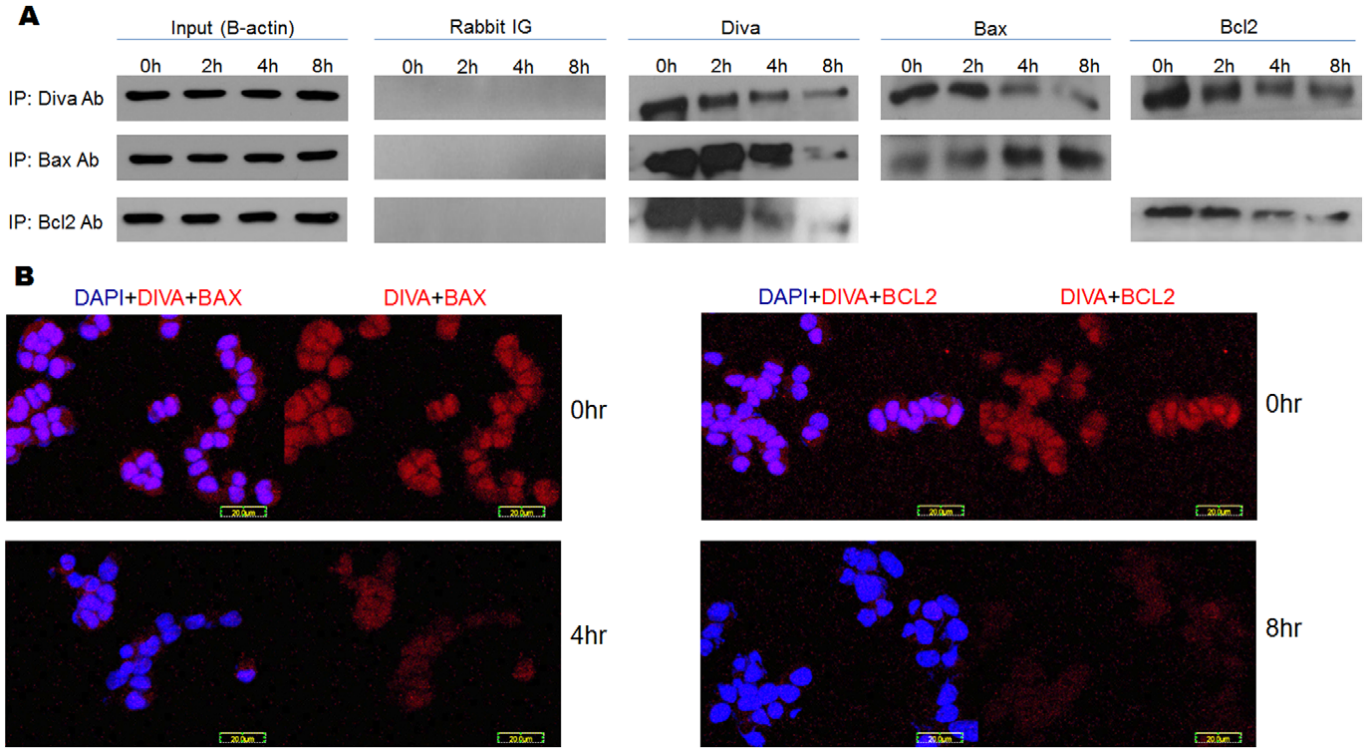
Bax and Bcl2 are associating partners of Diva. **a** Co-immunoprecipitation (Co-IP) and reverse immunoprecipitation of Diva, Bax and Bcl2 after 30 nM of H_2_O_2_ treatment. Total protein was isolated from treated PC12 cells after 0 hrs, 2 hrs, 4 hrs and 8 hrs of H_2_O_2_ treatment. Co-IP was first conducted with Diva antibodies to determine how Bax and Bcl2 interact with Diva during apoptosis. Reverse Co-IP was later conducted using Bax and Bcl2 antibodies to confirm the changes in protein binding. B-actin was used as an input control to ensure equal loading of total protein. Rabbit IG was used as a negative control to ensure no unspecific binding of IGs to antibodies occurred. Co-IP of Diva to B-actin was performed and used as a negative control (no bands shown). Pre-clearing of agarose beads was performed to ensure no unspecific binding of antibodies to beads occurred (no bands shown). **b** Duolink of Diva and Bax at 0 hr and 4 hr after H_2_O_2_ treatment to show the degree of interaction between Diva and Bax during apoptosis. **c** Duolink of Diva and Bcl2 at 0 hr and 8 hr after H_2_O_2_ treatment to show the degree of interaction between Diva and Bcl2 during apoptosis. Red Cy3 stained for interaction between two proteins while blue DAPI stained for the nucleus. Bar  = 20 µm.

Bcl2 family members dimerise or oligomerise to activate or inhibit each other. Through binding to effector pro-apoptotic proteins, anti-apoptotic members sequester them and prevent the downstream mitochondrial death cascade from activating [23], [24]. To identify the native binding partners of Diva and how these binding dynamics change with apoptosis, co-immunoprecipitation and duolink tests were performed. Following treatment with H_2_O_2_, the association of Diva with Bax and Bcl2 varied overtime. Co-immunoprecipitation carried out using Diva antibodies revealed decreasing interaction between Diva and Bax/Bcl2 (Fig. 2a). When reverse co-immunoprecipitation was done using Bax antibodies, decreased binding of Diva to Bax was still evident despite increasing Bax levels following oxidative stress. Similarly when reverse co-immunoprecipitation was carried out using Bcl2 antibodies, the association between Diva and Bcl2 was shown to decline (Fig. 2a). The decrease in binding of Diva to Bax was similar to previous studies carried out by Dlugosz who demonstrated a decline in Bcl2 interaction with Bax following apoptosis [25]. Despite the increase in Bax level, its binding to Diva still decreased as shown in the reverse co-immunoprecipitation done using Bax antibodies. This indicates a possible release of Bax from inhibition by Diva during apoptosis. Furthermore, given that the binding of Diva and Bcl2 decreased in a similar manner, it is possible that Diva co-operates with Bcl2 to control the activation of Bax during apoptosis. To reinforce the results, duolink staining was performed to observe the association of Diva with Bax and Bcl2. As interaction between 2 proteins gives rise to red fluorescence, an increase in the number of interactions would correspond to more fluorescence. Figures 2b reveals the degree of interaction between Diva and Bax, while figure 2c displays the interaction between Diva and Bcl2. Consistent with results from co-immunoprecipitation, the association between the proteins waned as apoptosis progressed.

### Knock Down of Diva Increases Mitochondrial Pathway Protein Expression and Mitochondrial Membrane Disruption

**Figure 3:**
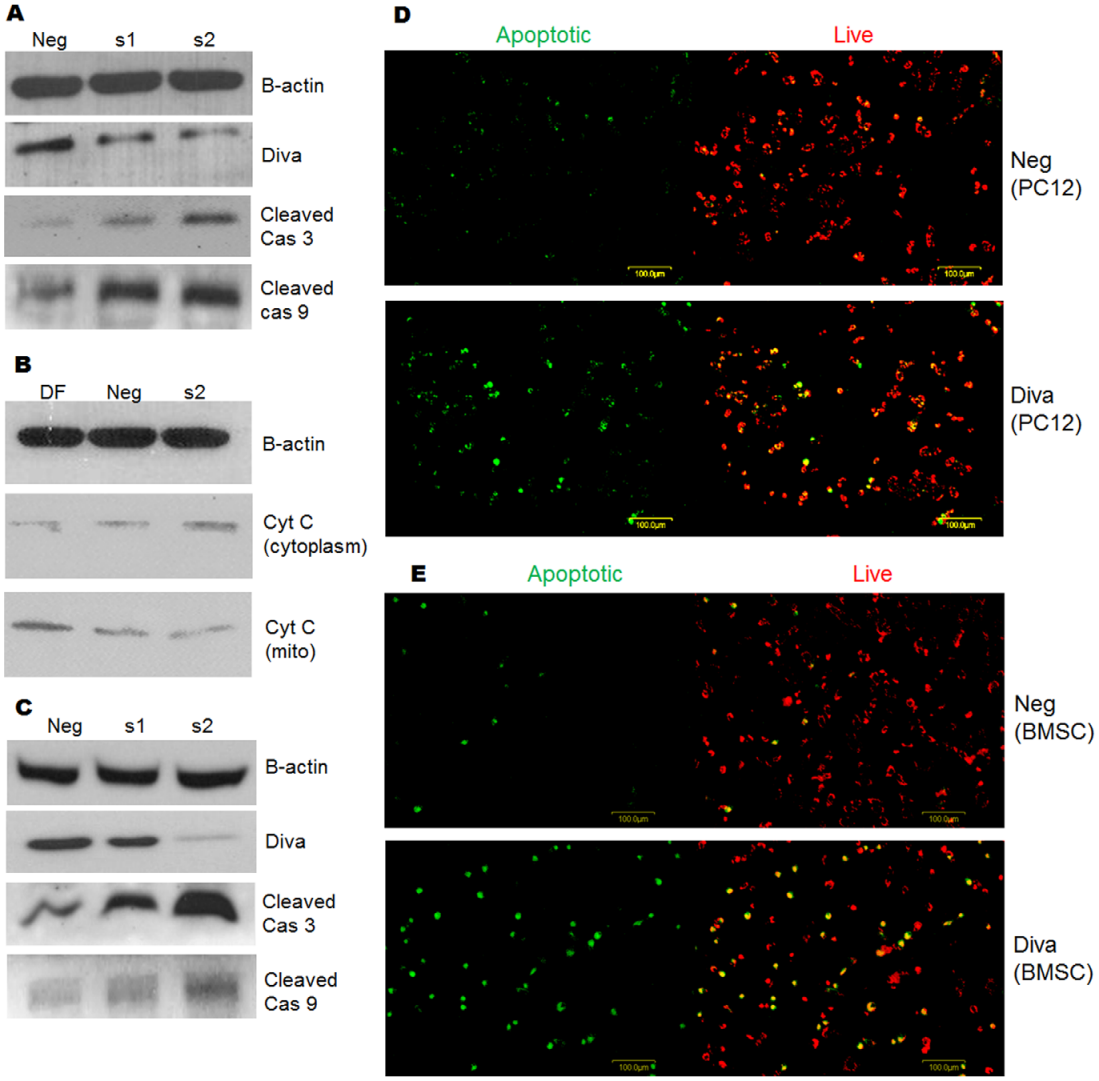
Knock down of Diva increases mitochondrial pathway protein expression and mitochondrial membrane disruption. **a–b** PC12 cells were harvested to determine the protein expression levels of Diva, cleaved caspase 9, cleaved caspase 3 and cytochrome C after Diva silencing. Western blot was conducted on the total protein extracted three days after Diva knock down. DF, Neg, s1 and s2 represent transfection reagent only, negative control siRNA, Diva siRNA1 and Diva siRNA2 respectively. **c** Western Blot showing protein levels of Diva, cleaved caspase 3 and cleaved caspase 9 three days after Diva silencing in BMSCs. B-actin was used as the loading control. **d** Mitochondrial membrane potential of PC12 was assessed by staining transfected cells with the JC-1 assay stain. The top panel shows PC12 which had been transfected with the negative control siRNA, while the bottom panel shows PC12 48 hrs after Diva silencing. Green stained for apoptotic cells with low mitochondrial potential while red stained for live cells with high mitochondrial potential. **e** JC-1 staining of BMSCs 48 hrs after transfection with negative control siRNA and Diva siRNA. The top panel shows BMSCs which had been transfected with the negative control siRNA, while the bottom panel shows BMSCs 48 hrs after Diva silencing. Bar  = 100 µm.

As shown earlier in Fig. 1, the expression of Diva mimics that of Bcl2 in the mitochondrial pathway. To ascertain both the function and signalling pathway of Diva, Diva siRNA was transfected into both PC12 and BMSCs. The western blot results in figures 3a, 3b and 3c reveal the increase in cleaved caspase 3, cleaved caspase 9 and cytochrome C protein expressions following successful Diva knock down. Notably, the endogenous expression of Diva was detected with 30 µg of BMSCs protein used to run the western blot. The fact that alterations in these proteins that lie in the mitochondrial pathway were seen, it confirms our earlier findings (Fig. 1) that Diva acts through the intrinsic mitochondrial death pathway. Activation of caspase 3 had been found to cause downstream cleavage of cytochrome C1 in the mitochondrial respiratory chain. This amplifies mitochondrial disruption and cytochrome C release [13]. To determine if the loss of Diva would also lead to mitochondrial disruption, the JC-1 reagent was employed. As demonstrated in figures 3d and 3e, the silencing of Diva in PC12 and BMSCs increased the number of green stained cells that corresponded to cells with low mitochondrial membrane potential (DCm). These indicate that the loss of Diva had enhanced mitochondrial disruption and membrane potential dissipation.

### Diva Silencing Increases the Proportion of Apoptotic Cells

**Figure 4:**
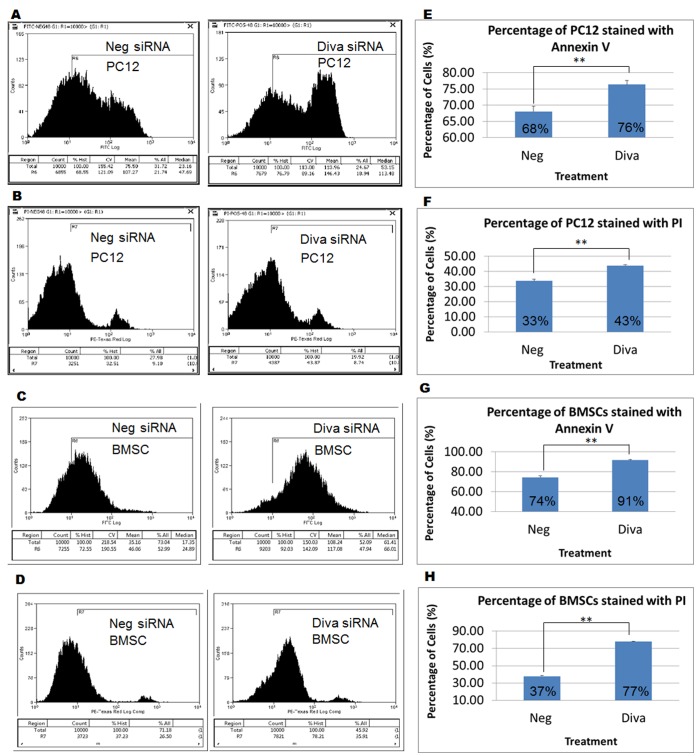
Diva silencing increases the proportion of apoptotic cells. **a** Flow cytometry performed on live PC12 cells within 1 hr of Annexin V staining. PC12 cells were stained 48 hrs after transfection with negative control and Diva siRNA. **b** Flow cytometry performed on live PC12 cells within 1 hr of PI staining. PC12 cells were stained 48 hrs after transfection with negative control and Diva siRNA. **c** Flow cytometry result of Annexin V stained negative control siRNA and Diva siRNA transfected BMSCs. **d** Flow cytometry result of PI stained negative control siRNA and Diva siRNA transfected BMSCs. Annexin V and PI staining were performed on live BMSCs 48 hrs after transfection. **e–f** Graphs showing the relative increase in Diva siRNA transfected PC12 cells that had taken up the Annexin V and PI stains respectively. **g–h** Graphs showing the relative increase in Diva siRNA transfected BMSCs that had taken up the Annexin V and PI stains respectively. Results were statistically significant with p-values <0.01.

To validate that the silencing of Diva would hasten apoptosis in cells, the Annexin V PI flow cytometric analysis was performed. Our results demonstrate obvious shift of graphs to the right following Diva siRNA treatment for 48 hrs (Fig. 4a–4d). These suggest that following the loss of Diva, more PC12 and BMSCs absorbed the Annexin V and PI stains as compared to negative siRNA control cells. In PC12 cells, 1.12 times more Diva siRNA transfected cells had taken up the Annexin V stain as compared to the negative control siRNA transfected cells. Similarly, 1.3 times more cells had taken up the PI stain following Diva siRNA treatment. In BMSCs, similar phenomenon was observed with 1.23 times more Diva siRNA transfected cells taking up the Annexin V stain as compared to the control cells. Flow cytometry of BMSCs stained with PI also showed 2.05 times more Diva siRNA treated cells taking up the PI stain (Fig. 4e–4h). Evidently, the silencing of Diva in both cell types had enhanced apoptosis in them.

### Loss of Diva Decreases Cell Number and Viability

**Figure 5:**
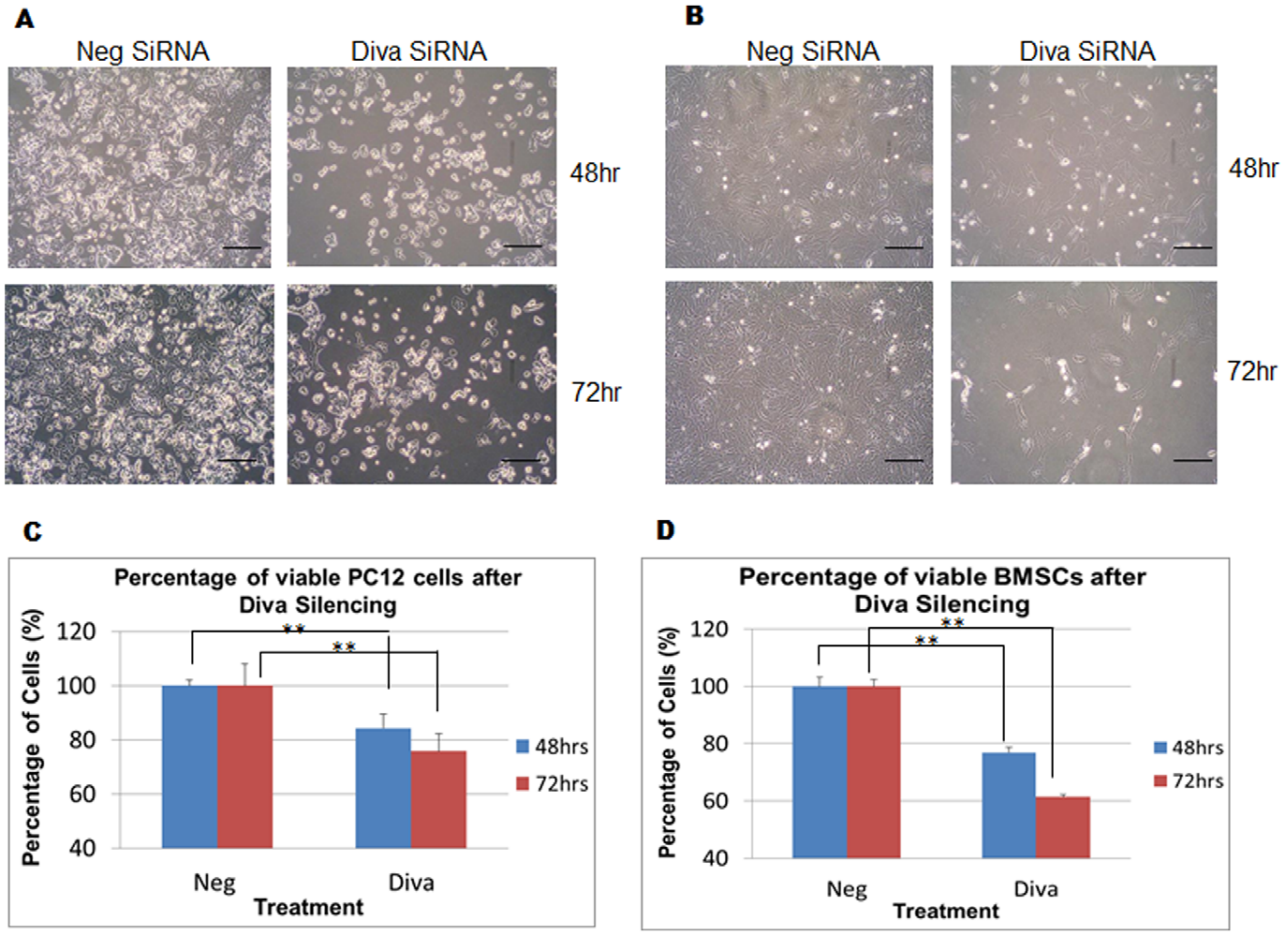
Loss of Diva decreases cell number and viability. **a** PC12 was transfected with both negative control siRNA and Diva siRNA to assess PC12 for changes in cell attachment to plate surface following Diva silencing. The top panel shows cells 48 hrs after transfection while the bottom panel shows cells 72 hrs after transfection. **b** Cell attachment images of BMSCs 48 hrs and 72 hrs after silencing. Bar  = 100 µm. **c** MTS assay was used to monitor the changes in PC12 cell number following transfection with negative control siRNA and Diva siRNA. Graph showing the percentage of viable PC12 cells in relation to the negative control after Diva silencing for 48 hrs and 72 hrs. Neg and Diva represent negative control siRNA and Diva siRNA2 respectively. **d** Graph showing the percentage of viable BMSCs in relation to the negative control after Diva silencing for 48 hrs and 72 hrs. Results were statistically significant with p-values <0.01.

Since the silencing of Diva enhanced mitochondrial pathway proteins, disrupted the DCm, and increased the proportion of apoptotic cells, we wanted to verify if these changes could be translated into decreased cell numbers. When the MTS assay was applied to Diva silenced cells, significant decreases in PC12 and BMSC viabilities were noted. In PC12 cells, the percentage of viable Diva silenced cells was reduced by 16% and 24% after 48 hrs and 72 hrs of transfection respectively (Fig. 5c). Similarly in BMSCs, the percentage of viable Diva silenced cells was reduced by 23% and 31% following 48 hrs and 72 hrs of transfection respectively (Fig. 5d). Physically, a decline in the number of cells left attached to the plate surface was also evident (Fig. 5a and 5b).

### Overexpression of Diva Decreases Apoptosis and Mitochondrial Membrane Disruption

**Figure 6:**
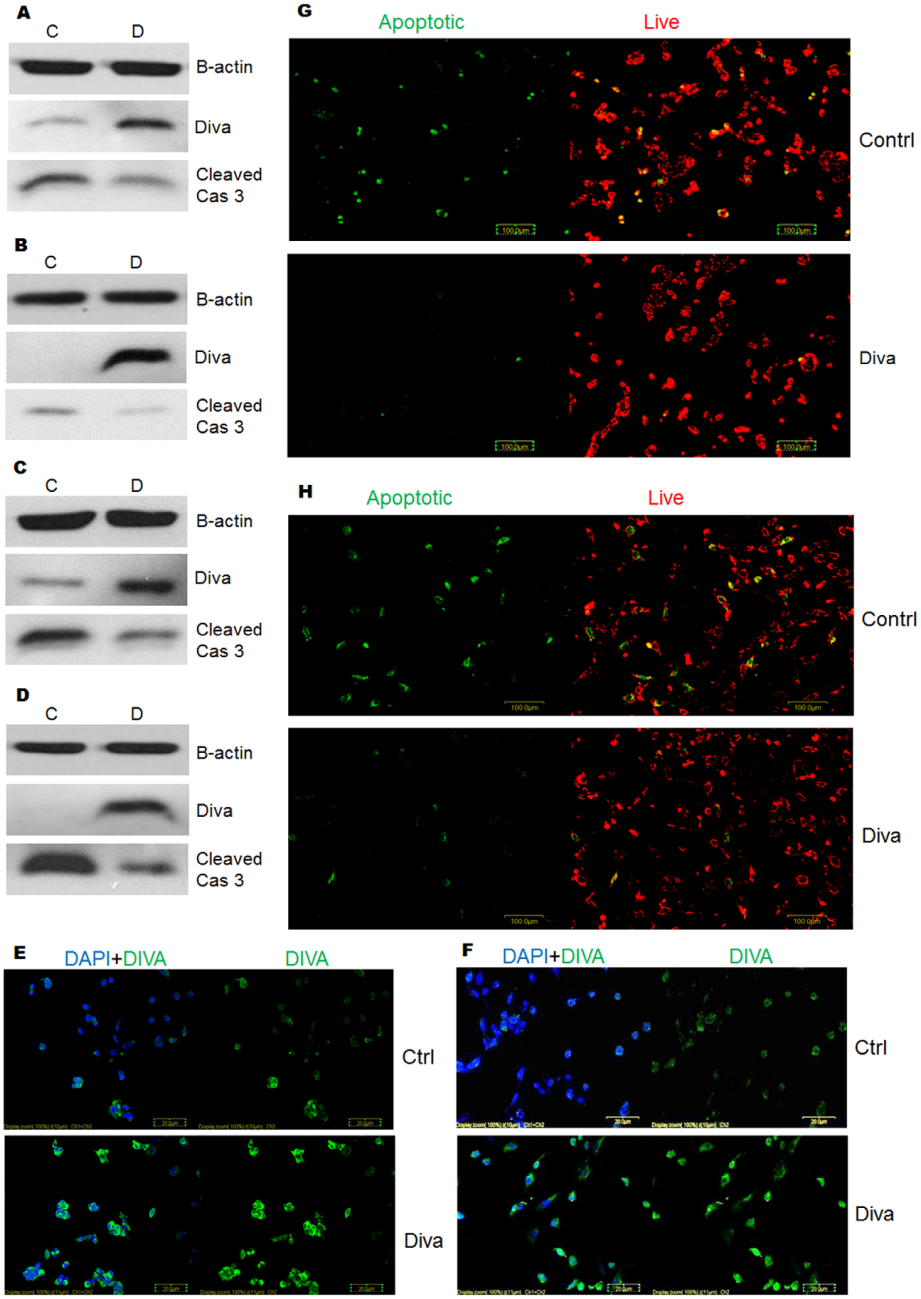
Overexpression of Diva decreases apoptosis and mitochondrial membrane disruption. **a** PC12 cells were harvested to determine the protein expression levels of Diva and Cleaved Caspase 3 after Diva overexpression. Western blot was conducted on the total protein extracted following transfection of PC12 cells with empty pcDNA6a control plasmids and Diva plasmids for 48 hrs. C represents control plasmid and D represents Diva plasmid. **b** Western blot of Diva and Cleaved Caspase 3 after overexpression of BMSCs with control and Diva plasmids for 48 hrs. **c** Total protein from PC12 cells were harvested to determine protein expression levels of Cleaved Caspase 3 following provocation of transfected cells with H_2_O_2_. Western blot was carried out on negative control and Diva plasmid transfected PC12 that were treated with 20 nM of H_2_O_2_ for 8 hrs. **d** Western blot after 6 hrs of H_2_O_2_ treatment on BMSCs that overexpress control and Diva plasmids. **e–f** Immunocytochemistry of PC12 and BMSCs 48 hrs after overexpression with control and Diva plasmids respectively. Green FITC stained for Diva while blue DAPI stained for the nucleus. Bar  = 20 µm. **g** Mitochondrial membrane potential of PC12 was assessed by staining transfected cells with the JC-1 assay stain. The top panel shows PC12 which had been transfected with the empty pcDNA6a vector control, while the bottom panel shows PC12 48 hrs after transfection with Diva plasmids. Green stained for apoptotic cells with low mitochondrial potential while red stained for live cells with high mitochondrial potential. **h** JC-1 staining of BMSCs 48 hrs after transfection with control and Diva plasmids. The top panel shows BMSCs which had been transfected with control plasmids, while the bottom panel shows BMSCs 48 hrs after transfection with Diva plasmids. Bar  = 100 µm.

Given that the silencing of Diva induced cell death, we next sought to determine the apoptotic effects of Diva overexpression on PC12 and BMSCs. Western blots probed with cleaved caspase 3 antibody were used to determine the degree of caspase 3 activation following Diva overexpression. As seen in figures 6a and 6b, the slight toxicity caused by the transfection reagent had caused some caspase 3 activation in PC12 and BMSCs respectively. However, the overexpression of Diva had evidently decreased caspase 3 processing in both cell types. Following provocation by 15 nM of H_2_O_2_, the difference in caspase 3 cleavage between empty vector and Diva transfected cells became even greater in both cell types (Fig. 6c and 6d). Strangely, the western blot was unable to detect endogenous levels of Diva in BMSCs but through immunocytochemistry (Fig. 6f) and PCR (not shown), endogenous Diva was evident. We therefore attributed this to the low BMSC protein concentration of less than 10 µg that was extracted as a result of the low BMSC numbers used for plasmid transfection. Following less caspase 3 activation, the overexpression of Diva also corresponded with a decline in the number of green stained cells with low DCm as shown in figures 6g and 6h. The overexpression of Diva in both PC12 and BMSCs had protected the cells from mitochondrial membrane potential transition caused by the slight toxicity of the transfection reagent.

### Diva Overexpression Enhances BMSC Protection Against Oxidative Stress and Microglial Toxicity

**Figure 7:**
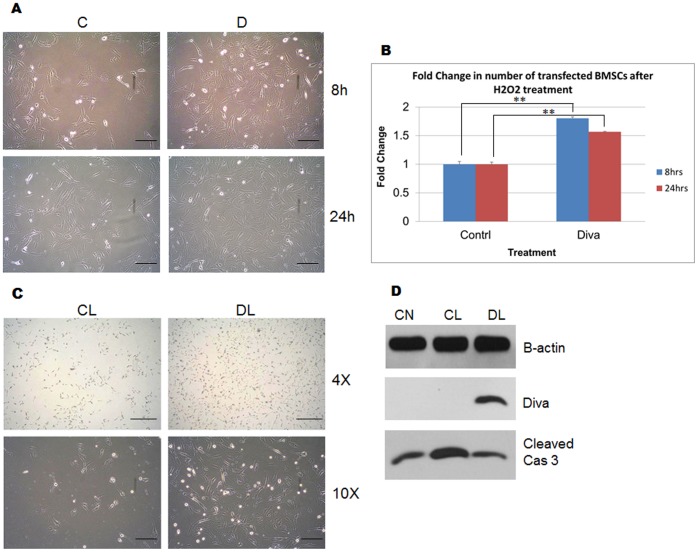
Diva overexpression enhances BMSC protection against oxidative stress and microglial toxicity. **a** BMSCs were transfected with empty vector control plasmids and Diva plasmids to assess BMSCs for changes in cell attachment to plate surface following treatment with H_2_O_2_. The top panel shows transfected cells 8 hrs after treatment with 20 nM of H_2_O_2_, while the bottom panel shows cells 24 hrs after treatment. C and D represent BMSCs overexpressed with control and Diva plasmids respectively. Bar  = 100 µm. **b** MTS assay was used to monitor the changes in transfected BMSC number following treatment with 20 nM of H_2_O_2_. Graph showing the fold change of Diva plasmid transfected BMSC number in relation to the negative control after H_2_O_2_ treatment for 8 hrs and 24 hrs. Results were statistically significant with p-values <0.01. **c** Cell attachment images of transfected BMSCs 24 hrs after treatment with LPS activated BV2 supernatant. CL represents control plasmid transfected cells with LPS activated BV2 supernatant. DL represents Diva plasmid transfected cells with the supernatant. Images taken at 4× and 10× magnifications. Bar  = 200 µm and 100 µm respectively. **d** Total protein from BMSCs was harvested to determine protein expression levels of Cleaved Caspase 3 following provocation of transfected cells with LPS activated BV2 supernatant. CN represents control plasmid transfected BMSCs in serum free DMEM. CL represents control plasmid transfected cells with LPS activated BV2 supernatant. DL represents Diva plasmid transfected cells with the supernatant.

Since the overexpression of Diva was able to reduce caspase 3 activation and protect BMSCs against DCm dissipation, we were next interested to find out if the overexpression of this protein could protect BMSCs against cell loss in response to oxidative stress. After treatment with H_2_O_2_, greater numbers of BMSCs which overexpressed Diva were left attached to the plate surface after 8 and 24 hrs (Fig. 7a). In addition, the MTS assay result exhibited significantly greater number of viable BMSCs overexpressing Diva after H_2_O_2_ provocation for various lengths of time. As shown in figure 7b, approximately 1.8 and 1.6 times more BMSCs overexpressing Diva were resistant to 8 hr and 24 hr exposures to oxidative stress. Furthermore, we were also interested to verify if BMSCs overexpressing Diva could resist cell death caused by microglial cytotoxicity. Judging by the outcome shown in figure 7c, it is apparent that the overexpression of Diva in BMSCs allowed more cells to remain attached to the plate surface. Intriguingly, the level of cleaved caspase 3 had even been brought down to levels similar to that of the untreated plasmid control (Fig. 7d).

## Discussion

Our group has taken interest in a relatively confusing Bcl2 family member called Diva. Till this day, little is known about this protein as a result of its specificity within limited tissues [9], [13]. Several groups had attempted to overexpress Diva in various cell lines, only to show conflicting results pertaining to its apoptotic role [10], [11]. To study the apoptotic function of Diva, we utilised PC12 and rat BMSCs which were found to express endogenous Diva protein. Given that Diva could be found in BMSCs, it raises a novel possibility that Diva might play an important role in controlling the survival of these stem cells. As mentioned earlier, MSCs possess functional apoptotic pathways that get activated after cell differentiation [8]. We therefore hypothesized that the changes in Diva expression could control the survival of our mesenchymal stem cells isolated from the bone marrow of *Sprague–Dawley* rats.

To first understand the mechanism of Diva’s actions, recombinant rat Fas ligand and H_2_O_2_ were used to induce apoptosis in PC12 cells. Fas ligand activates the extrinsic receptor apoptotic pathway through its interaction with the Fas receptor on the cell surface. The resultant interaction recruits FADD and causes autoactivation of procaspase 8, leading to cleavage of procaspase 3 into its active form [26], [27]. Results obtained from real time PCR and western blot showed no evident change in endogenous Diva, Bcl2, or Bax expressions following caspase 8 and 3 activation (Fig. 1a and 1d). However, oxidative stress caused by H_2_O_2_ produced a strong increase in the transcription and translation of Bax. Diva on the other hand showed significant downregulation that coincided with the change in Bcl2 (Fig. 1b, 1c and 1e). Activation of procaspase 9 and 3 following the dip in Bcl2/Bax ratio is typical of mitochondrial apoptotic pathway activation. This change in Bcl2/Bax ratio had also been previously described in PC12 oxidative stress models [28]. Since a similar change in the Diva/Bax ratio was seen, it not only suggests Diva’s participation in the intrinsic mitochondrial pathway, but it also indicates a possible anti-apoptotic role for Diva.

In addition to studying the apoptotic role of Diva, we were also interested to ascertain its native interacting partners. Previous exogenous overexpression of Diva within 293T cells yielded conflicting conclusions pertaining to Diva’s binding with Bcl2 and Bax [9], [11], [14]. To verify this, we induced apoptosis in PC12 cells before co-immunoprecipitation was carried out to identify the possible binding partners of Diva. At the same time, we went on to monitor how the binding dynamics of these proteins change with apoptosis. The results obtained in figure 2 illustrated decreased binding of Bax and Bcl2 to Diva as apoptosis progressed under oxidative stress. It has been established that the ability of Bcl2 to homodimerise or heterodimerise with other Bcl2 family members contributes significantly to its apoptotic activity. By binding to another anti-apoptotic protein, Bcl2 acquires a conformation that allows it to inhibit Bax, keeping Bax in the “globular" structure. This globular structure prevents Bax from oligomerising and forming mitochondrial channels. However when Bcl2 returns to its “globular" structure due to BH3 only protein binding, it releases Bax which can now form the “dagger" structure required for mitochondrial membrane insertion [29]. Previous studies done by Dlugosz demonstrated that Bcl2 interacts with Bax to prevent Bax oligomerisation at the mitochondria surface. Once an apoptotic stimulus is received, Bcl2 changes conformation to release Bax from sequestration [25]. The induction of Bcl2 structural change has been attributed to its binding with BH3 only proteins such as Bid and Bim [30]. Such binding of Diva to BH3 only proteins such as Harakiri had also been established recently [31]. Judging from our results, we postulate that through co-operation with Diva, Bcl2 is able to sequester Bax as shown by the strong initial binding of Diva to Bax. As apoptosis is triggered under oxidative stress, Diva releases both Bcl2 and Bax, thereby allowing Bax to amplify the apoptotic cascade.

Indeed, the loss of Diva in both PC12 and BMSCs enhanced apoptosis. With the successful silencing of Diva in PC12 (Fig. 3a), significant increases in cytochrome C, cleaved caspase 9 and cleaved caspase 3 were observed (Fig. 3a and 3b). This surge in cytochrome C release and activation of the mitochondrial pathway could possibly be due to the release of Bax by Diva as shown by the co-immunoprecipitation and duolink (Fig. 2a and 2b). In conjunction with declining mitochondrial membrane potential (DCm), increased Annexin V/PI dye uptake and decreased cell viability (Fig. 3, 4 and 5), we have reason to believe that the loss of Diva drives the fate of cells towards cell death. Such downregulation of anti-apoptotic Bcl2 members had been previously reported to cause disturbance to the mitochondrial membrane potential [32]. With the loss of Bcl2, mitochondrial membrane integrity can no longer be maintained as Bax oligomerises to form pores in the membrane [33]. The silencing of Bcl2 had also been shown to enhance cells’ susceptibility towards oxidative stress and irradiation [34], [35]. Like PC12 cells, BMSCs were highly prone to caspase cleavage, mitochondrial instability and apoptotic cell loss following Diva downregulation. The fact that such grave apoptosis was witnessed indicates the importance of Diva in preventing cell death in BMSCs. Interestingly, such cell death in the developing nervous system and ovaries was not prominent in Diva null mice which displayed no growth or fertility abnormalities. Moreover under ionizing radiation, the rate of apoptosis that occurred in the ovaries of Diva null mice was no different from the wild type [36]. Similar discrepancies were noted in the small intestines of Bcl2 null mice which revealed no difference in apoptotic rates from the wild type mice after gamma radiation exposure. In contrast with the cells from the small intestines, colonic stem cells exhibited dramatic apoptosis after exposure to radiation [37], [38]. Clearly, the effect of a Bcl2 member knock out was tissue and cell type dependent.

To confirm the importance of Diva in cell survival, the overexpression of Diva in PC12 and BMSCs was carried out to evaluate its anti-apoptotic ability. With obvious reductions in cleaved caspase 3 expression following Diva’s upregulation in PC12 and BMSCs, it is therefore indicative of Diva’s ability to resist cell death. Moreover, this overexpression of Diva was able to protect cells against DCm dissipation. As mentioned earlier, the oligomerisation and insertion of Bax into the mitochondrial membrane releases cytochrome C and causes subsequent disruption to the membrane integrity. With the overexpression of Diva, it is possible that such membrane disruption is prevented as a result of Bax sequestration. Such overexpression of Bcl-xL has also been previously reported to prevent DCm reduction induced by oxygen deprivation and growth factor withdrawal [39], [40]. In addition to preventing cell losses in response to oxidative stress, the overexpression of Diva in BMSCs was also able to reduce caspase 3 cleavage and loss of cells in response to LPS induced BV2 microglial conditioned medium (Fig. 7). LPS has been shown to activate microglial cells to produce a myriad of pro-inflammatory molecules such as TNF-α, IL-1b, IL-6 and iNOS [41], [42]. This mimics the responses of activated microglial cells which migrate into lesion sites and make the environment unfavourable for transplanted BMSC survival [43]. Our results therefore suggest the possibility of overexpressing Diva in BMSCs to increase the success rates of stem cells therapy.

In short, the results obtained in this study have shown that Diva plays an important anti-apoptotic role in preventing cell death through the mitochondrial pathway. It does so possibly by co-operating with Bcl2 to sequester Bax, thereby preventing mitochondrial induced death. In order to confirm this point, large-scale mutagenesis of Diva binding domains will be carried out. Given that the silencing of Diva in BMSCs had caused a surge in cell death, while the overexpression of this protein conferred strong protection, it gives us reason to believe that Diva plays a crucial role in regulating BMSC survival.

## Materials and Methods

### Apoptotic Treatment

Recombinant Rat Fas Ligand (FasL) (F0552, Sigma, USA) and Hydrogen Peroxide (H_2_O_2_) (H3410, Sigma, USA) were used to induce apoptosis in PC12 cells (CRL-1721, ATCC, USA). PC12 were first grown in 6 well plates at a density of 0.5×10^6^ cells/ml. Thereafter, the cells were treated with 100 ng/ml of FasL for 48 hrs. To induce oxidative stress in cells, 30 nM of H_2_O_2_ was added to PC12 grown at a density of 0.8×10^6^ cells/ml. These cells were treated for 8 hrs to induce apoptosis.

### RNA Extraction, cDNA Conversion and Real Time PCR

To study the changes in Diva, Bcl2 and Bax RNA expressions after drug treatment, the RNA from treated PC12 cells was extracted and converted to cDNA. RNA extraction was done using the RNeasy mini kit (74106, Qiagen, Germany), following the manufacturer’s protocol. Both the concentration and purity of resultant RNA were tested using the Nanodrop machine. Following this, 2 µg of RNA was then converted to cDNA with the Promega (USA) kit which contains Oligo dT primers (C1101), dNTP (U1515), RNase inhibitor (N2511) and M-MLV reverse transcriptase (M1705). The converted cDNA was then used as the template to run real time PCR in the LightCycler 2.0 instrument (Roche, Germany). Rat Diva, Bcl2, or Bax primers (1st Base, Singapore) were mixed with the cDNA template, SYBRE green master mix (204163, Qiagen, Germany) and RNase free PCR buffer for each reaction. GAPDH primers (1st base, Singapore) were used to study this endogenous control.

### Co-immunoprecipitation

To investigate the identity of Diva’s associating partners, co-immunoprecipitation was carried out on PC12 protein extracts obtained after the induction of cell death by H_2_O_2_. Proteins were extracted from PC12 cells with M-PER Mammalian Protein Extraction Reagent (78501, Pierce, USA), EDTA, and protease inhibitor (78430, Pierce, USA). Protein concentrations were first measured using the Bradford assay (500-0201, Bio-Rad, USA), before the concentration in each time point was standardised in all tubes. To investigate if Bcl2 and Bax associate with Diva, 200 µl of protein sample from each time point was mixed with 2 µl of each (Diva, Bcl2 or Bax) antibody (Cell signalling, USA). The mixture was left to shake overnight at 4°C. The next day, 20 µl of Protein G Plus/Protein A Agarose Suspension (IP05, Calbiochem, Germany) was added into each tube and shook for 4 hrs at 4°C. The tubes of suspension were later washed 5 times in washing buffer (10 mM Tris; Adjust to pH 7.4, 1 mM EDTA, 1 mM EGTA; pH 8.0, 150 mM NaCl, 1% Triton X-100, 0.2 mM sodium ortho-vanadate and protease inhibitor cocktail) and mixed with 10 µl of 6× loading dye. Samples were heated at 100°C for 5 mins prior to western blotting. A pre-clearing step was conducted to ensure that no unspecific binding of Diva, Bcl2 or Bax proteins to the agarose beads occurred. Furthermore, B-actin and rabbit IG (Cell signalling, USA) were used as the input and negative controls respectively for the experiments.

### Duolink

To validate the results found using co-immunoprecipitation, the Duolink kit I (Olink Bioscience, Sweden) was used. PC12 cells were plated onto 12 well plates overlaid with coverslips. 0.25×10^6^ cells were added into each well and grown for 48 hrs. Following this, 20 nM of H_2_O_2_ was used to treat the cells. The cells were washed with 1× PBS and fixed in 4% paraformaldehyde for 15 mins. Each well was blocked with 5% horse serum for 1 hr at room temperature, before addition of Diva (SC8740, Santa Cruz, USA) and Bax/Bcl2 (2772/2876, Cell Signalling, USA) antibodies at 4°C overnight. The next day, the Duolink kit was applied according to the manufacturer’s protocol. The DAKO fluorescent medium (S3023, DAKO, Denmark) mounted slides were analysed with the Olympus confocal microscope with XYZ axis stacking.

### Rat Bone Marrow Mesenchymal Stem Cell Isolation

To obtain rat BMSCs for the study of Diva, 10 mg/ml of 5-fluorouracil (F8423, Sigma, St Louis, MO) was injected intraperitoneally into one-month-old male *Sprague–Dawley* rats weighing 120–150 g. The drug was administered at 80 mg/kg two days before the rats were sacrificed. Bone marrow cells were then harvested from the femurs and tibias of the rats [44]. Centrifugation of bone marrow aspirates with Sigma Histopaque (density 1.077) was performed to separate mononuclear cells from the bone marrow extracts. The aspirates were then diluted with PBS at a ratio of 10∶17 (aspirate: PBS). The diluted cell suspension was first carefully layered onto the interface of the Histopague at a ratio of 1∶1 (histopaque: cell aspirate), before centrifuging the cells at 400×g for 30 minutes at 24°C. After centrifugation, the mononuclear cells found as the middle opaque interface were extracted and washed twice in 10 ml of PBS. At each washing step, the cells were centrifuged at 250×g for 10 minutes to remove all traces of Histopaque present in the suspension. The pellet of mononuclear cells was resuspended in DMEM supplemented with 10% Fetal Calf serum, 10% New Born Calf Serum (Invitrogen) and 1% penicillin-streptomycin solution (Sigma). Thereafter, the cells were grown in T-25 cm^2^ culture flasks at a density of approximately 500 000 cells/cm^2^. The unattached cells were removed via medium exchange 48 hrs after plating, and adherent cells were left to grow in the flask for 10–14 days with media replacement every third day.

### Silencing Transfection

To study the effects of Diva knock down on PC12 and BMSCs, Diva siRNA and Negative control siRNA (Dharmacon, USA) were used. The two Diva siRNAs, S1 and S2, target the following distinct sequences in rat Diva mRNA: 5′- GTTAAGCGGAGGAGGGAT -3′ and 5′- CGGATGAGTTGCTCTCCAA -3′ respectively. The ON-TARGET plus Non-targeting siRNA (D-001810-0X, Dharmacon, USA) was used as a negative control for the experiments. PC12 and BMSCs were seeded into 6 well plates for 24 hrs at densities of 0.25×10^6^ and 0.2×10^6^ cells/ml respectively. The next day, 20 µM of siRNA and 3 µl of Dharmafect 2 (T-2002-03, Dharmacon, USA) were diluted in serum free DMEM for 5 mins before mixing the 2 reagents at room temperature for 20 mins. Following that, the mixture was then added to the wells containing DMEM supplemented with 10% FBS. A complete medium change was performed 24 hrs post transfection.

### Overexpression Transfection

To observe the effects of Diva overexpression on PC12 and BMSCs, a Diva plasmid construct was synthesized (Blue Heron, U.S.A) and cloned into pcDNA6a vector (V221-20, Invitrogen, USA). PC12 and BMSCs were seeded into 6 well plates for 24 hrs at densities of 0.35×10^6^ and 0.08×10^6^ cells/ml respectively. Thereafter, transfection was carried out with both empty pcDNA6a control and Diva plasmids. 2.5 µg of plasmid and 2 µl of Lipofectamine 2000 (11668-019, Invitrogen, USA) were added into separate tubes of DMEM OPTI-MEM (11058-021, Invitrogen, USA) for 5 mins. Thereafter, the 2 reagents were mixed and incubated for 15 mins at room temperature. The mixture of plasmid and Lipofectamine was then applied to the wells containing DMEM supplemented with 10% FBS. Plates were incubated at 37°C and 5% CO_2_ before a complete medium change was performed after 6 hrs.

### Mitochondrial Protein Extraction

To monitor the changes in cytochrome C protein distribution following Diva siRNA knock down, the Mitochondria Isolation kit (89874, Pierce, USA) was used. After silencing Diva in PC12 cells, cytosolic proteins and the mitochondria were isolated according to the manufacturer’s protocol. Following that, the isolated mitochondria were washed with 0.5 M sodium chloride before lysing of the mitochondria with 2% CHAPS (28300, Pierce, USA) in TRIS buffered saline (28379, Pierce, USA). The concentration of the final mitochondrial protein extract was measured with the BCA Protein Assay (23227, Pierce, USA).

### Western Blot

Proteins extracted from PC12 and/or BMSCs were subjected to western blot analysis. The changes in various protein levels were detected with anti-Diva (3869), anti-Bcl2 (2876), anti-Bax (2772), anti-cleaved caspase 8 (9429), anti-cleaved caspase 9 (9507), anti-cleaved caspase 3 (9664), anti-cytochrome C (4280) (Cell Signalling, USA) and anti-B-actin (A5441, Sigma, USA) antibodies. The cells were lysed using M-PER Mammalian Protein Extraction Reagent (78501, Pierce, USA), EDTA, and protease inhibitor (78430, Pierce, USA). To plot the standard curve for protein concentration testing, a protein standard was obtained through serial dilutions of Bovine Serum Albumin (500-0002, Bio-Rad, USA). The concentration of extracted proteins were then tested with the Bradford assay (500-0201, Bio-Rad, USA) and compared against the standard curve. Following this, test samples were diluted with 6× loading dye before running western blot in 10% SDS polyacrylamide gels. The gels were later electro-transferred for 1 hr 15 mins onto polyvinylidene difluoride (162-0176, Bio-Rad, USA) membrane using a Semi-dry transfer cell (Trans-blot SD, Bio-Rad). The PVDF membrane strips were blocked with 5% nonfat milk in 0.1% Tween-20 TBS (TTBS) on a shaker for 1 hr, before the addition of primary antibodies at 4°C overnight. The next day, the membranes were washed in 1×TTBS before secondary HRP-conjugated antibodies (7074, Cell Signalling, USA) were added for 1 hr at room temperature. Another 3 washing steps were done and the immunocomplexes were observed by applying a chemiluminescent substrate (34075, Pierce, USA) to the membranes for 5 mins in the dark. Protein bands were visualized after X-ray exposure and the optical density of each band was then measured using the NIH ImageJ software.

### Immunocytochemistry

Fluorescent staining was performed on PC12 and BMSC samples after Diva overexpression. The cells were stained for Diva (SC8740, Santa Cruz, USA) at a concentration of 1∶200. Secondary antibodies conjugated to FITC (AP106F, Millipore, Germany) were used at a concentration of 1∶300. The cells in 6 well plates were first fixed with 4% paraformaldehyde for 15 mins, before permeabilisation with 1×PBSTX for 15 mins. Blocking was then done with 5% horse serum for 1 hr at room temperature. Following that, primary antibodies were added overnight at 4°C. The next day, cells were washed and secondary antibodies were added for 1 hr at room temperature. DAPI (D9542, Sigma, USA) was then added for 5 mins at a concentration of 1∶4000, before final washing was performed. Coverslips were later mounted on glass slides with fluorescent mounting medium (S3023, DAKO, Denmark) and analysed with the OLYMPUS confocal microscope with XYZ axis stacking.

### Mitochondrial Membrane Potential Change Detection

To determine if overexpression and silencing of Diva in PC12 and BMSCs would cause variations in the mitochondrial membrane potential, the JC-1 kit (10009172, Cayman, USA) was utilised. 48 hrs after overexpression or silencing of Diva in 6 well plates, 800 µl of FBS free DMEM was first added into each well. Thereafter, 20 µl of the JC-1 Assay reagent was mixed into each well and incubated for 25 mins at 37°C in the dark. By the end of 25 mins, the medium was discarded and wells were washed twice with 1×PBS. Fresh 1×PBS was applied to the wells to ensure that cells do not dry up as fluorescent images were taken within 30 mins. Images were taken with the Olympus confocal microscope and XYZ stacking was performed.

### MTS Assay

To monitor the changes in cell number and viability of PC12 and BMSCs after transfection and drug treatments, the CellTitre Aqueous One Solution Cell Proliferation Assay (G3581, Promega, USA) was used on cells grown in 6 well plates. To attain the standard curve for PC12 cells, 0.05×10^6^, 0.1×10^6^, 0.2×10^6^, 0.4×10^6^, 0.8×10^6^ and 1.6×10^6^ cells/ml were added into each well. BMSCs were plated at increasing concentrations of 0.02×10^6^, 0.04×10^6^, 0.08×10^6^, 0.16×10^6^, 0.32×10^6^ and 0.64×10^6^ cells/ml. After 4 hrs of settling, 400 µl of MTS mixture was added into each well and incubated for 2 hrs at 37°C and 5% CO2 in the dark. The optical density of each well was then read and recorded by the Genios plate reader (Tecan, Germany). Two plots of cell number against optical density were drawn and used as standards. Once the standard curves had been determined, 400 µl of MTS reagent was dissolved into the culture medium of treated cells and incubated in the dark for 2 hrs at 37°C and 5% CO2. Following that, the optical density of each well was read by the Genios plate reader at 490 nm and compared against the standard curve to determine the cell number.

### BV2 Microglial Cell Stimulation and Supernatant Collection

The BV2 cell, an immortalized mouse microglial cell line that exhibits the morphological and functional characteristics of microglia, was a gift from Dr MJ Strong [45]. BV2 cells were grown till 90% confluence in 75 cm^2^ flasks for 1 day. Thereafter, 1 µg/mL of Lipopolysaccharide (L2637, Sigma, USA) was added into the flask to activate the BV2 cells for 24 hrs. The next day, cells were washed twice with 1×PBS before fresh FBS free DMEM was added into the flask. The supernatant was collected, centrifuged and filtered through a 0.2 um filter (16532K, Sartorius, France) at the end of 24 hrs before storage at −80°C.

### Flow Cytometry with Annexin V and Propidium Iodide

To check if the silencing of Diva caused apoptosis in both PC12 and BMSCs, the FITC Annexin V Apoptosis Detection Kit I (556547, BD Pharmingen, USA) was used. The transfected cells were first typsinised with trypsin and neutralised with DMEM. Thereafter, cells were centrifuged, washed twice in ice cold 1×PBS, and centrifuged again at 2000 rpm for 5 mins at 4°C. Cells were resuspended in 100 µl of 1× binding buffer, before addition of 5 µl of FITC Annexin V or 5 µl of PI. Following the 15 mins incubation at room temperature, another 400 µl of 1× binding buffer was added into each sample. Samples were analysed by flow cytometry within 1 hr.

### Statistics

All experiments were repeated at least three times for statistical analysis. The student’s T-Test was applied and a p-value of less than or equal to 0.05 was used to determine the level of significance. The mean standard deviation was also included where appropriate.
